# A Prospective Observational Study on the Clinical and Microbiological Profile of Catheter-Related Blood Stream Infections in Hemodialysis Patients and Their Outcome in a Tertiary Care Centre in Northeast India

**DOI:** 10.7759/cureus.99983

**Published:** 2025-12-23

**Authors:** Gayatri Pegu, Angelia L Khawbung, Manjuri Sharma, Prodip K Doley, Miranda Pegu

**Affiliations:** 1 Department of Nephrology, Gauhati Medical College and Hospital, Guwahati, IND

**Keywords:** catheter-days, catheter-related bloodstream infection, central venous catheter, gram-negative bacilli, incidence, mortality

## Abstract

Introduction: Catheter-related bloodstream infections (CRBSIs) are major complications for hemodialysis patients dependent on central venous catheters (CVCs). Variations in infection control practices and prolonged catheter use amplify the risk and lead to increased morbidity, mortality, and healthcare costs.

Objectives: This study aimed to determine the clinical and microbiological profile of CRBSIs in hemodialysis patients and to assess the outcomes, including in-hospital mortality.

Methodology: This prospective observational study was conducted in the Department of Nephrology, Gauhati Medical College, in Guwahati, Assam, India, from May 2024 to April 2025. A total of 67 adult patients (≥18 years) undergoing maintenance hemodialysis with tunneled or non-tunneled CVCs and clinically suspected CRBSIs were enrolled. CRBSI was defined using the Centers for Disease Control and Prevention (CDC) and Infectious Diseases Society of America (IDSA) criteria. Patients with alternative identifiable infection sources or incomplete microbiological data were excluded. Baseline demographics, comorbidities, clinical presentation, laboratory parameters, catheter characteristics, microbiological profile, treatment, and in-hospital outcomes were obtained from medical records using a standardized data collection form. Blood cultures were obtained aseptically prior to antibiotic initiation, including paired peripheral and catheter-drawn samples. Samples were processed using the BACTEC system, and organism identification was performed using the VITEK 2 system in accordance with standard microbiological protocols.

CRBSI incidence was calculated as episodes per 1,000 catheter-days. Clinical outcomes assessed included catheter removal, length of hospital stay, and all-cause in-hospital mortality. Continuous variables were expressed as mean ± standard deviation or median (interquartile range), and categorical variables as frequencies and percentages. Univariate comparisons were performed using Student’s t-test for continuous variables and Chi-square or Fisher’s exact test for categorical variables, as appropriate. Variables with a p-value <0.10 on univariate analysis were entered into the multivariate logistic regression model. A p-value <0.05 was considered statistically significant. Statistical analyses were performed using IBM SPSS Statistics.

Results: The mean age was 48.2 ± 12.8 years, and 62.7% of patients were male. Diabetes and hypertension were the leading causes of end-stage kidney disease (ESKD) (44.8%), indicating a high-risk cohort with prolonged catheter exposure. Total catheter exposure was 6,645 days, with a CRBSI incidence of 10.08 episodes per 1,000 catheter-days. Blood cultures were positive in 74.6% of cases, yielding Gram-positive cocci (34.8%), Gram-negative bacilli (39.0%), and fungi (1.5%), while 25.4% remained culture-negative. In-hospital mortality was 31.3%. Non-survivors showed higher rates of Gram-negative infection (55.0% vs. 36.2%; p = 0.04) and culture positivity (85.0% vs. 70.2%; p = 0.08). Median catheter dwell time was similar between groups (1.5 months each; p = 0.17).

Conclusion: The observed CRBSI incidence and mortality exceeded rates reported in high-resource settings. Early arteriovenous fistula creation, strict adherence to aseptic protocols, and empiric antimicrobial regimens covering both Gram-positive and Gram-negative organisms are urgently required.

## Introduction

Catheter-related bloodstream infections (CRBSIs) are a serious complication in patients undergoing hemodialysis, especially those dependent on central venous catheters (CVCs) [1]. CRBSI is defined, according to the Centers for Disease Control and Prevention (CDC) and the Infectious Diseases Society of America (IDSA) criteria, as bacteremia or fungemia occurring in a patient with an intravascular catheter, in which the catheter is identified as the source of infection based on microbiological evidence and in the absence of an alternative identifiable focus. Compared to arteriovenous fistulas (AVFs), CVCs are associated with higher infection rates and more adverse outcomes [2].

Mahmood et al. (2024) observed that CRBSIs affect nearly 44% of patients with CVCs [3]. However, this report comes from a single-centre study and may not be generalizable to other settings. Brown et al. (2018) and Miller et al. (2016) reported a 7.5-fold greater risk of infection compared to AV access [4,5]. However, these variations are attributable to differences in study designs and outcome definitions. Several studies noted that these infections could lead to prolonged hospitalization, increased morbidity, and mortality rates that reach up to 25% [6-8]. The dilemma is particularly pronounced in lower and middle-income nations, where constrained resources and erratic infection control measures may exacerbate the associated risks. Zakaria et al. (2021) and Ravani et al. (2013) highlighted the disproportionate burden in such settings [9,10]. 

Microbiologically, *Staphylococcus aureus *(including methicillin-resistant strains) is the predominant pathogen along with *Staphylococcus epidermidis *and other coagulase-negative staphylococci. Although Gram-positive cocci remain predominant in most large series (40-80% of cases), Gram-negative organisms are not negligible, particularly in low-resource settings where they may even outnumber Gram-positives [11-13]. Gram-negative organisms such as *Pseudomonas aeruginosa* and *Klebsiella *species are also increasingly reported in regions with variable antibiotic stewardship [14]. Factors such as inadequate adherence to aseptic protocols and insufficient training among dialysis personnel are significant contributors to the incidence of CRBSIs. Chen et al. (2019) and Mahmood et al. (2024) highlighted these gaps regarding CABSI incidence [3,15]. Preventive strategies such as antimicrobial lock therapy and catheter-care innovations have been documented but remain inconsistently implemented [15].

Gram-negative bacilli represent a relatively minor fraction of total cases of CRBSIs (approximately 20-40%). However, their influence on patients undergoing hemodialysis is far from negligible. Wouters and colleagues documented in 2018 that *Pseudomonas aeruginosa, Klebsiella pneumoniae, *and *Escherichia coli *collectively account for a substantial share of Gram-negative infections, with extended‐spectrum β‐lactamase production increasingly observed [16]. Pandit et al. (2024) studied hemodialysis patients in Northeast India and found that high antibiotic resistance made it harder to identify effective empirical drugs and hence was linked to longer hospital stays [17].

CRBSIs pose a significant challenge within the realm of hemodialysis practice. In accordance with the CDC 1991 definition and IDSA 2009 guidelines, CRBSI diagnosis requires positive blood cultures (central or peripheral) in the absence of another infectious focus [18]. The Infectious Diseases Society of America (IDSA), based on the CDC framework, insists that isolating identical pathogens from catheter-tip cultures and peripheral blood specimens strengthens diagnostic confidence [18]. The National Kidney Foundation’s KDOQI elaborated that clinical signs (fever or chills) with concordant central and peripheral cultures - for CRBSI identification in patients whose symptomatology overlaps with chronic kidney disease manifestations [19].

Geographic disparities in CRBSI rates elucidate variations in catheter utilization practices and infection-control protocols. Temporary and non‐tunneled catheters persist as a weak link: Weldetensae et al. (2023) reported that these devices can incur infection rates as high as 10.8 episodes per 1,000 catheter‐days, as compared with 0.55-4.4 for cuffed, tunneled alternatives [20].

India’s hemodialysis programs continued to grapple with comparatively high CRBSI burdens. Agrawal et al. (2019) documented 7.4 episodes per 1,000 catheter‐days among patients with jugular non‐tunneled CVCs [21]. This high rate clearly indicates gaps in training, protocol adherence, and access to long‐term vascular planning. Preventive strategies such as antimicrobial locking solutions have demonstrated efficacy as observed in a study by Sheng et al. (2020); they observed a decrease in CRBSI incidence by over 50% in randomized trials [22]. These studies underscore the necessity for tailored strategies in the prevention and management of CRBSIs within the context of hemodialysis. Employing (1) standardized diagnostic criteria, (2) meticulous tracking of infection rates, (3) vigilant monitoring of pathogens, and (4) the implementation of targeted preventive measures significantly mitigates the risk of infection.

Our study meticulously examined the clinical characteristics, including patient demographics, comorbidities, type and duration of vascular access, presenting clinical features, and relevant laboratory parameters, as well as the microbiological characteristics, comprising the spectrum of causative organisms isolated from blood cultures and their antimicrobial susceptibility patterns, of CRBSIs among hemodialysis patients, and evaluated their outcomes, including treatment response, catheter management, associated complications, length of hospital stay, and in-hospital mortality, at a tertiary care facility in Northeast India.

## Materials and methods

Study design and setting

This was a single-centre prospective observational study conducted at the Department of Nephrology, Gauhati Medical College, a tertiary care centre in Guwahati, Assam, India. The study was conducted from May 2024 to April 2025. The study was designed to evaluate the clinical and microbiological profile of CRBSIs in hemodialysis patients and their outcome.

Study population and sampling

All patients receiving maintenance hemodialysis were invited to participate in the study. Inclusion criteria were subjects on maintenance hemodialysis for more than three months, patients with vascular access with clinical and laboratory findings of CRBSI as per CDC/IDSA criteria, age more than 18 years, patients of both sexes, and willingness to participate in the study.

Patients with a documented infection within the preceding one month were excluded to avoid misclassification from persistent or partially treated bacteremia and to ensure that BSIs met the CDC/IDSA criteria for catheter-related origin rather than reflecting a pre-existing infection. Unwillingness to give consent was also excluded from the study population.

Data collection

Patient demographics, comorbidities (e.g., diabetes mellitus, hypertension), catheter type (temporary double‐lumen jugular catheter vs. permanent tunneled cuffed catheter), and catheter insertion and removal dates were abstracted from electronic medical records. Clinical features at presentation, i.e., vital signs, laboratory parameters (white blood cell count, CRP), and antibiotic therapy details were recorded. Outcomes were classified as “Cured” (resolution of clinical and microbiological infection without in‐hospital mortality) or “Expired” (death during the same hospitalization).

Microbiological Methods

Blood cultures were performed using the automated BACTEC system (Becton Dickinson, USA). For each blood culture, 10 mL of blood was inoculated per bottle. Two sets were drawn per episode (one from the catheter hub and one from a peripheral vein). The hub was disinfected using 0.5% chlorhexidine in alcohol before sampling. Bottles were incubated for up to 5 days using an automated culture system. Contamination was judged based on clinical correlation. Positive cultures were sub-cultured on blood agar and MacConkey agar. Organism identification and antibiotic susceptibility testing were conducted using the VITEK 2 Compact system (bioMérieux, France). 

Outcome Measures

The primary outcome was in‐hospital mortality (“Expired”) versus clinical cure (“Cured”). Secondary outcomes included (a) Incidence rate of CRBSI per 1,000 catheter-days, calculated as - (Number of CRBSI episodes x 1000 / Total catheter days at risk). (b) Distribution of causative organisms (Gram-positive, Gram-negative, fungal).

Empirical antibiotic therapy was initiated at the time of clinical suspicion of CRBSI, based on local microbiological patterns and institutional antibiotic policy. Therapy was later modified according to culture and sensitivity results. Catheters were removed in the presence of persistent fever or bacteremia beyond 48-72 hours of appropriate antibiotic therapy, hemodynamic instability, tunnel or exit-site infection, evidence of metastatic infection, or infection due to *S. aureus*, *Pseudomonas*, fungi, or other highly virulent organisms. In patients with limited vascular access, catheter salvage was attempted only if clinical response was satisfactory and blood cultures became sterile with appropriate therapy.

Statistical Analysis

Data were analyzed using IBM SPSS Statistics for Windows, version 29.0 (released 2022, IBM Corp., Armonk, NY). Normally distributed data presented as mean ± standard deviation (SD); non‐normal data as median (interquartile range [IQR]). Categorical variables were expressed as frequencies and percentages. Univariate analysis was performed with (a) continuous variables, i.e., Student’s t-test or Mann-Whitney U test, as appropriate, and (b) categorical variables, i.e., Chi-square test or Fisher’s exact test. 

Multivariate analysis was conducted with variables that had p < 0.10 on univariate testing, and they were then entered into a logistic regression model to identify independent predictors of mortality. A p-value threshold of < 0.10 was used as a screening criterion to avoid excluding potentially important predictors at the univariate stage. Adjusted odds ratios (aOR) with 95% confidence intervals (CIs) were reported. Adjusted odds ratios were calculated using multivariable logistic regression, with adjustment for clinically relevant and a priori-selected confounders, including age, sex, presence of diabetes and hypertension, duration of catheter dwell time, type of dialysis catheter (temporary vs. tunneled), culture positivity, and infecting organism category (Gram-positive vs. Gram-negative), as these factors are known to influence the risk and outcomes of CRBSIs. A two-tailed p < 0.05 was considered statistically significant.

## Results

Baseline demographics

A total of 67 confirmed cases of CRBSI patients were included in this study. The mean age was 48.2 ± 12.8 years with a male predominance (62.7% male vs. 37.3% female).

Underlying causes of end-stage kidney disease (ESKD) in hemodialysis patients with CRBSI: The most common cause of ESKD was diabetes mellitus (44.8%), followed by hypertension (32.8%). Less frequent etiologies included IgA nephropathy (3.0%), and various other causes (each 1.5%) - congenital anomalies of the kidney and urinary tract (CAKUT), autosomal-dominant polycystic kidney disease, and nephronophthisis.

**Figure 1:**
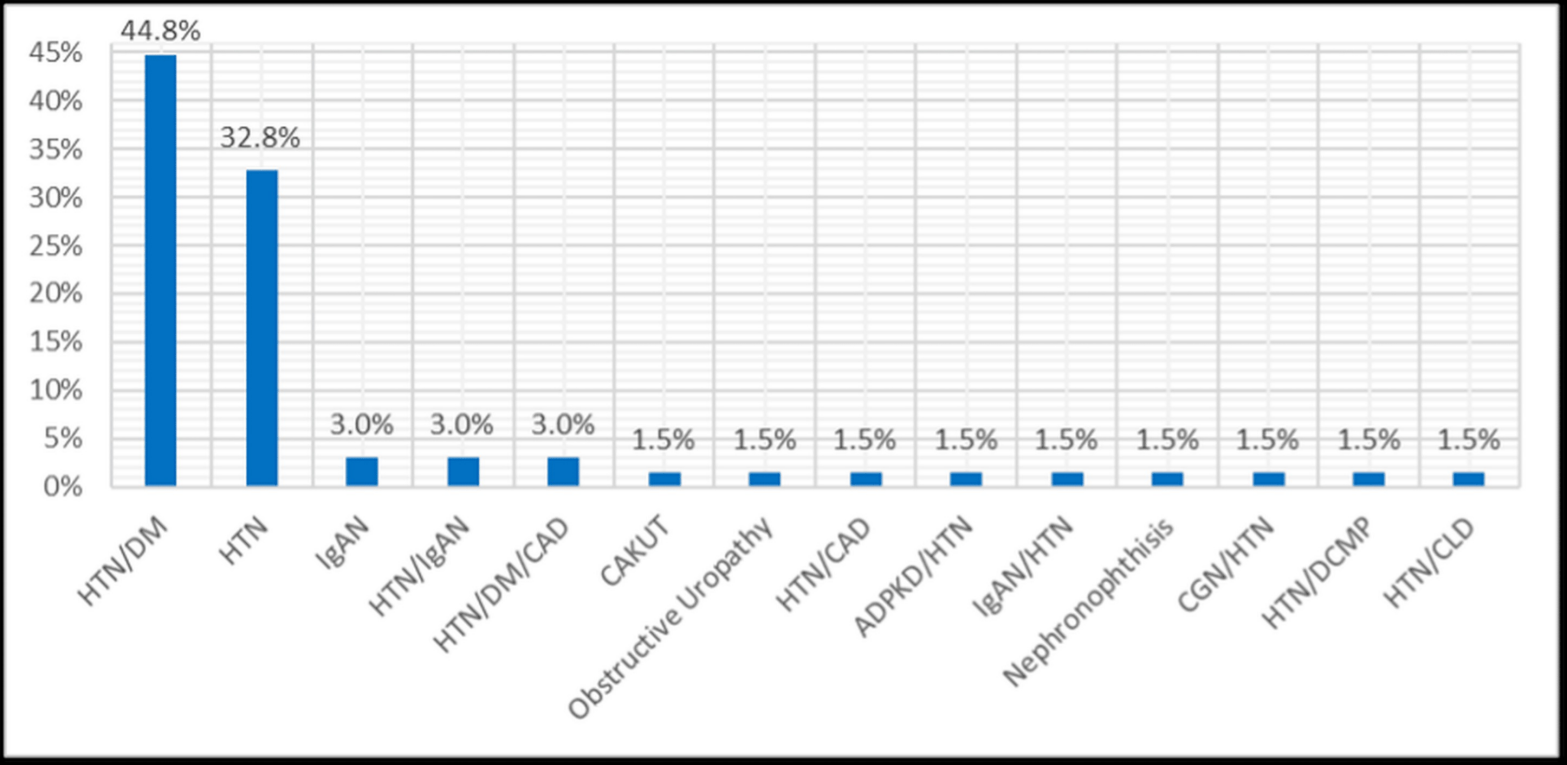
Distribution of primary diseases (%). ADPKD, autosomal dominant polycystic kidney disease; CAD, coronary artery disease; CAKUT, congenital anomalies of the kidney and urinary tract; CGN, chronic glomerulonephritis; CLD, chronic liver disease; DCMP, dilated cardiomyopathy; DM, diabetes mellitus; HTN, hypertension; IgAN, Ig A nephropathy Figure created by the authors with MS Excel (Microsoft Corp., USA)

The distribution of primary diseases is shown in Figure 1.

Microbiological profile

Blood cultures were found to be positive in 74.6%. Blood cultures yielded no growth in 25.4% of cases. Among positive cultures, *Staphylococcus aureus* (19.4%) and coagulase-negative staphylococci (10.4%) were the highest Gram-positive pathogens. Gram-negative bacilli accounted for a substantial proportion of isolates: *Klebsiella pneumoniae *(7.5%), *Pseudomonas* *aeruginosa* (6.0%), and* Escherichia coli *(6.0%) being the most common. 

We noted lesser contributions from *Enterobacter* spp. and *Acinetobacter *spp., fungal growth (*Candida albicans*), and rare pathogens such as *Burkholderia cepacia *complex and *Stenotrophomonas maltophilia*. This spectrum indicated both skin-derived Gram-positive organisms and the burden of Gram-negative and opportunistic agents in this tertiary-care setting.

**Figure 2:**
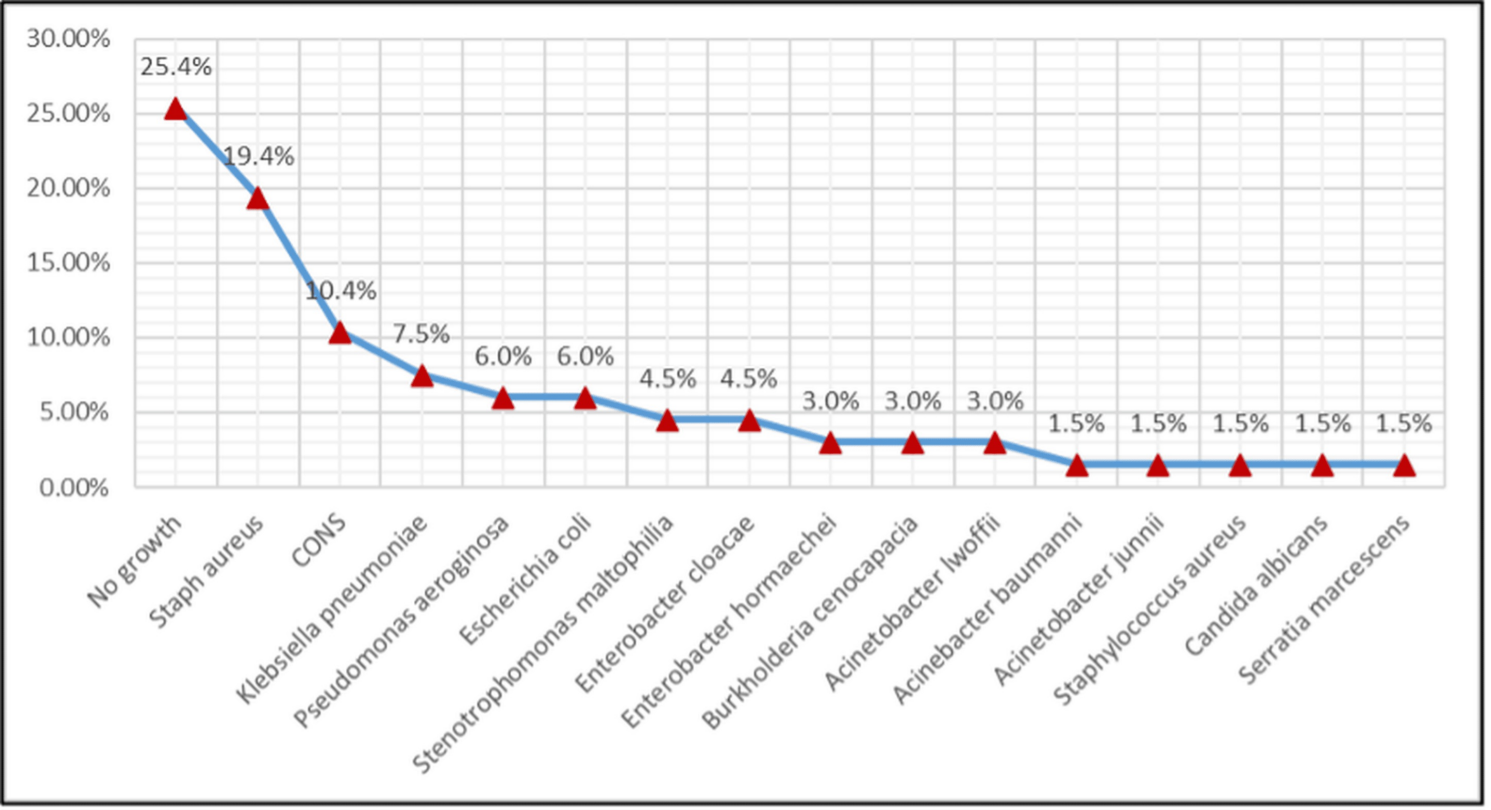
Microbiological profile of catheter-related bloodstream infection isolates in hemodialysis patients CONS, Coagulase-negative staphylococci Figure created by the authors with MS Excel (Microsoft Corp., USA)

Figure 2 shows the microbiological profile of CRBSI isolates in hemodialysis patients.

Univariate analysis

Patients who expired were slightly older (mean 50.5 ± 10.9 years vs. 47.2 ± 13.6 years) and had a higher proportion of males who expired (55.0% vs. 66.0%) compared to those who were cured.

The univariate comparison of clinical and microbiological characteristics by outcome is summarized in Table 1.

**Table 1:** Univariate comparison of clinical and microbiological characteristics by outcome DLJC, double-lumen jugular catheter; SD, standard deviation

Characteristics	Cured	Expired
Mean age ± SD (years)	47.2 ± 13.6	50.5 ± 10.9
% Male	66.0%	55.0%
% Temporary catheter (DLJC)	53.2%	45.0%
% Culture-positive	70.2%	85.0%
% Gram-positive isolates	34.0%	25.0%
% Gram-negative isolates	36.2%	55.0%

The expired group had fewer temporary (DLJC) catheters in situ (45.0% vs. 53.2%) but a higher rate of culture-positive infections (85.0% vs. 70.2%). Gram-negative organisms were more prevalent among the expired cohort (55.0% vs. 36.2%). Gram-positive isolates predominated in the cured group (34.0% vs. 25.0%). These differences suggested associations between patient age, catheter type, and organism profile with mortality in CRBSIs.

Multivariate logistic regression analysis for predictors of mortality: In the logistic regression model adjusting for age, sex, catheter type, culture positivity, and organism category, no variable reached statistical significance as an independent predictor of in-hospital mortality. Trend-wise, culture-positive infections were associated with over a twofold higher odds of death (adjusted OR 2.16, 95% CI 0.45-10.28; p = 0.333).

Table 2 outlines the multivariate analysis of factors associated with mortality in CRBSI patients.

**Table 2:** Multivariate analysis of factors associated with mortality in CRBSI patients CI, confidence interval; DLJC, double-lumen jugular catheter

Variable	Unadjusted OR (95% CI)	Unadjusted p-value	Adjusted OR (95% CI)	Adjusted p-value
Age (per year increase)	1.02 (0.98–1.07)	0.345	1.03 (0.98–1.07)	0.266
Male sex (vs. female)	0.63 (0.22–1.83)	0.398	0.65 (0.21–2.01)	0.457
Temporary catheter (DLJC vs. Permacath)	0.88 (0.31–2.51)	0.811	0.89 (0.30–2.62)	0.835
Culture-positive infection (vs. no growth)	2.40 (0.61–9.53)	0.212	1.96 (0.41–9.22)	0.396
Gram-negative isolate (vs. non–Gram-negative)	1.76 (0.61–5.09)	0.293	1.34 (0.40–4.54)	0.633

Unadjusted and adjusted ORs were estimated using logistic regression with mortality as the dependent variable. "Culture-positive" was defined as any growth versus no growth. "Non-Gram-negative" included gram-positive isolates, fungal isolate(s), and no-growth cultures (reference group).

Gram-negative isolates were associated with higher odds of mortality, but this was not statistically significant (adjusted OR 1.34, 95% CI 0.40-4.54; p = 0.633). Temporary DLJC access and male sex showed point estimates below 1.0, suggesting lower odds of death (adjusted OR 0.89 and 0.65, respectively), although both estimates were imprecise with wide confidence intervals and non-significant p-values (p = 0.835 and p = 0.457). Increasing age showed a small, non-significant association with mortality (adjusted OR 1.03 per year, 95% CI 0.98-1.07; p = 0.266). None of the evaluated clinical or microbiological variables was observed to be an independent predictor of mortality in this cohort.

Catheter dwell-time comparison by patient outcome: Catheter dwell-times were comparable between patients who were cured and those who expired. In the cured group, the median dwell-time was 2.0 months (IQR: 1.5-4.0 months).

**Figure 3:**
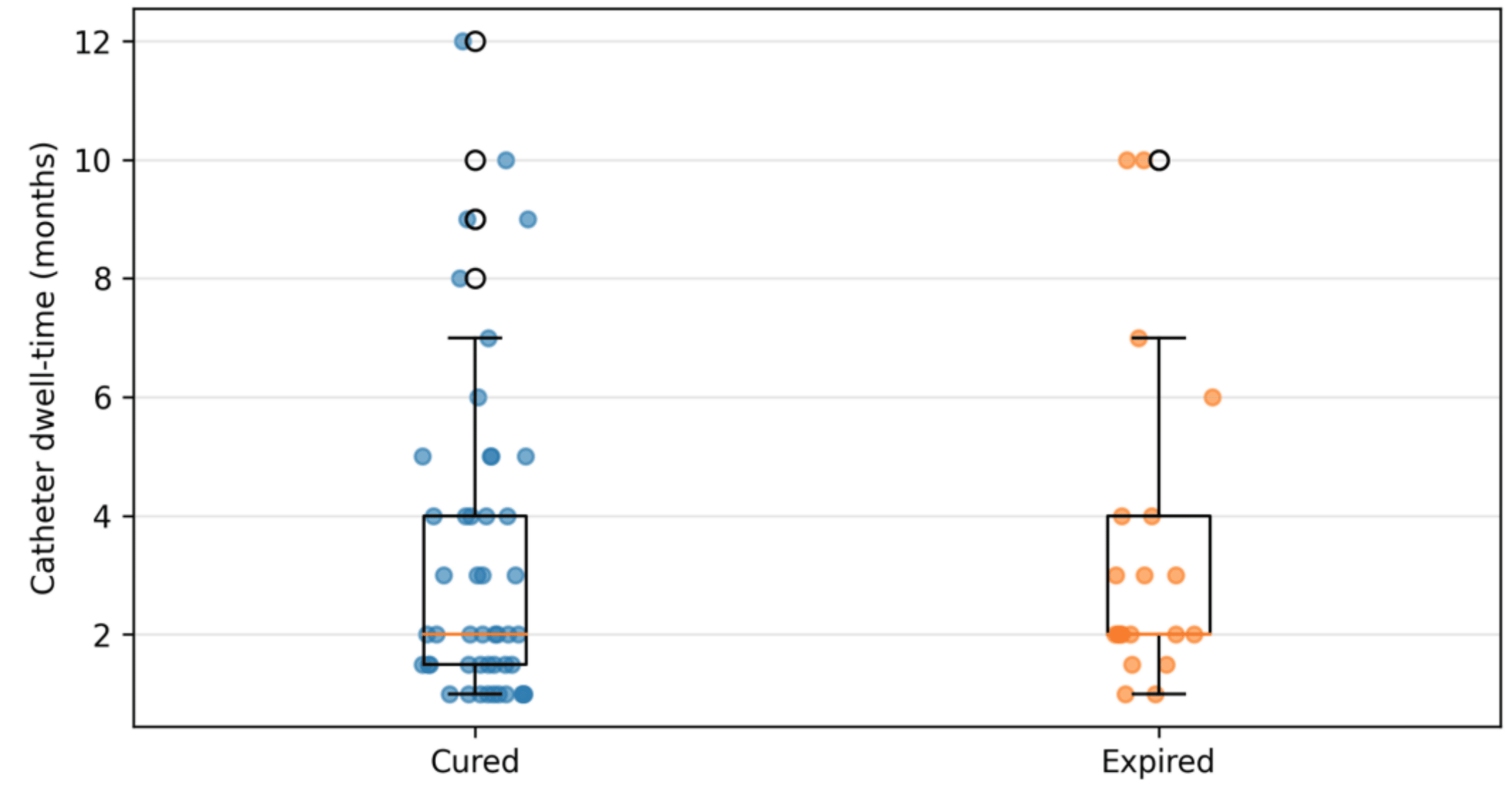
Catheter dwell time by outcome catheter dwell time by outcome Figure created by the authors with MS Excel (Microsoft Corp., USA)

Figure 3 shows the catheter dwell-time by outcome.

In the expired group, the median was also 2.0 months (IQR: 2.0-4.0 months). A two-sided Mann-Whitney U test showed no statistically significant difference in catheter dwell times between groups (U = 413.0, p = 0.442). This indicated that catheter duration did not differ significantly between survivors and non-survivors in this cohort. Note: Dwell-time analyses and organism grouping were based on cultures obtained at the index CRBSI presentation. All patients had evidence of single pathogens throughout the study period. There was no polymicrobial growth found.

CRBSI Incidence per 1,000 Catheter-Days: Across the study cohort, 67 CRBSI episodes occurred over a total of 6,645 catheter-days at risk. 

Table 3 outlines the incidence of CRBSI per 1,000 catheter-days.

**Table 3:** Incidence of CRBSIs per 1,000 catheter-days Note: Total catheter-days at risk were computed as the sum of catheter dwell-time (in days) for all included patients/catheters during the observation period up to catheter removal/exchange, discharge, death or end of follow-up (as applicable). Incidence was expressed per 1,000 catheter-days. CRBSIs: catheter-related bloodstream infections

Metric	Value
Number of CRBSI episodes included	67
Total catheter-days at risk	6645
Incidence per 1000 catheter-days	10.08

Accordingly, the incidence was CRBSI episodes per 1,000 catheter-days, providing a standardized measure that can be compared with other hemodialysis cohorts.

Association of Microbial Category With Patient Outcomes

When stratified by microbial category, the single patient with a fungal isolate succumbed (100% mortality), although the sample is too small for firm conclusions. Among Gram-negative infections, 39.3% (11/28) of the patients expired, compared with 23.8% (5/21) in the Gram-positive group and 17.6% (3/17) among culture-negative cases.

Table 4 shows the outcome stratified by microbial category.

**Table 4:** Outcome stratified by microbial category Outcome stratified by microbiological category (index CRBSI episode). Percentages are row percentages within each microbiological category. CRBSI: catheter-related bloodstream infection

Category	Cured	Expired	Total (n)
Fungal	0 (0.0%)	1 (100.0%)	1
Gram-negative	17 (60.7%)	11 (39.3%)	28
Gram-positive	16 (76.2%)	5 (23.8%)	21
No growth	14 (82.4%)	3 (17.6%)	17
Total	47	20	67

Table 4 contextualizes the multivariable findings by presenting unadjusted outcome proportions by pathogen category and demonstrating the small numbers within some strata that limit statistical power. It is included to provide an easily interpretable descriptive breakdown of outcomes by pathogen category, complementing Table 3 by showing crude outcome proportions and highlighting small stratum sizes (e.g., fungal), which contribute to wide confidence intervals. Statistically significant association was not observed with a chi-square test between organism category and outcome (χ² = 5.12, p = 0.164). The microbial category alone was not a clear predictor of mortality in our study population.

## Discussion

In this single-centre cohort of 67 hemodialysis patients with CRBSIs, we observed a predominantly middle-aged population (mean age 48.2 ± 12.8 years) with male preponderance (62.7%). Hypertension with diabetes mellitus was the most common underlying renal diagnosis in our cohort. Collectively, our data showed the substantial cardiometabolic burden among patients reaching ESKD and requiring catheter-based hemodialysis in our setting.

The overall CRBSI incidence was 10.08 episodes per 1,000 catheter-days, calculated from 67 CRBSI episodes over 6,645 catheter-days at risk. This rate is higher than what is typically reported in high-resource dialysis programmes, and it underscores the need to strengthen catheter-care bundles, staff training, and early planning for permanent vascular access at centres facing resource and workflow constraints.

Microbiologically, cultures were positive in 74.6% of episodes, with a substantial Gram-negative burden (39.0%) alongside Gram-positive cocci (34.8%) and rare fungal infection (1.5%); 25.4% of episodes were culture-negative. The relatively high proportion of Gram-negative pathogens in particular has important empirical-treatment implications in centres where resistant *Enterobacterales* and non-fermenters are prevalent, and supports using initial regimens that cover both Gram-positive and Gram-negative organisms.

Clinical outcomes were concerning, with an in-hospital mortality of 31.3%. On univariate comparison, non-survivors had higher culture positivity (85.0% vs. 70.2%) and a higher proportion of Gram-negative isolates (55.0% vs. 36.2%). However, in multivariable logistic regression adjusting for age, sex, catheter type, culture positivity, and organism category, none of the included variables independently predicted mortality. Culture positivity showed higher point estimates for death compared with no growth (adjusted OR 1.96, 95% CI 0.41-9.22), and Gram-negative etiology also trended toward higher mortality (adjusted OR 1.34, 95% CI 0.40-4.54), but both associations were non-significant, likely reflecting limited power and imprecision.

Catheter dwell-time did not differ by outcome: the median dwell-time was 2.0 months in both cured and expired groups (IQR 1.5-4.0 vs. 2.0-4.0 months), and the Mann-Whitney U test showed no significant difference (U = 413.0, p = 0.442). This suggests that, within our cohort, catheter duration alone was not a discriminating predictor of mortality once CRBSI occurred, highlighting the likely influence of host factors, severity of illness at presentation, and timeliness/appropriateness of therapy.

When outcomes were stratified by microbiological category, mortality was 39.3% in Gram-negative infections (11/28), 23.8% in Gram-positive infections (5/21), and 17.6% in culture-negative episodes (3/17); the single fungal case was fatal. While these descriptive patterns are clinically intuitive, the small numbers, especially for fungal infection-limit inference, and organism category alone were not a clear predictor of mortality in this dataset.

Weldetensae et al. (2023) conducted a retrospective cross-sectional study from January 2016 to June 2022 at Ayder Comprehensive Specialized Hospital, Mekelle University [20]. They enrolled 353 hemodialysis patients with central venous catheters (CVC) in situ for more than 48 hours, meticulously collecting sociodemographic, clinical, microbiological, and catheter-related data. The incidence was recorded at 7.74 CRBSI episodes per 1,000 catheter-days, with Gram-negative isolates constituting 57.6% of the infections; notably, *Escherichia coli* emerged as the predominant Gram-negative pathogen. Subsequent multivariate logistic regression analysis revealed that catheter duration of less than 30 days served as a protective factor (AOR 0.30; p < 0.001), while a history of previous CVC infection was identified as a significant risk factor (AOR 11.9; p < 0.001). Furthermore, residing in urban areas was associated with an increased risk of CRBSI (AOR 1.92; p < 0.05), and a hemoglobin level below 12 mg/dl heightened this risk (AOR 2.78; p < 0.05). Conversely, a leukocyte count of 10,000 cells/mm³ or fewer demonstrated a protective effect (AOR 0.31; p < 0.001). The authors concluded that proactive planning for arteriovenous (AV) fistulas and the implementation of empirical treatment regimens targeting Gram-negative organisms and *Staphylococcus aureus* are strongly recommended [20]. Our study, by contrast, reported a higher CRBSI incidence (10.08 vs. 7.74 episodes per 1,000 catheter-days). Both cohorts demonstrated a prominent Gram-negative burden, though this was greater in their study (57.6%) than in ours (39.0%). *While Escherichia coli *predominated in their cohort, we observed a wider Gram-negative spectrum, including *Klebsiella* and *Pseudomonas* species. Unlike their identification of catheter duration and anemia as risk factors for CRBSI occurrence, catheter dwell time did not influence mortality in our cohort. 

Shah et al. (2020) conducted a retrospective cohort study from 2014 to 2018 at the dialysis unit of a private tertiary care hospital, Mumbai [23]. They followed patients for a total of 109,929 catheter-days and recorded 40 CRBSI episodes. Incidence rate was 0.36 per 1,000 catheter-days, and distribution was equal between temporary and permanent catheters (20 episodes each). Forty-two organisms were isolated from 40 episodes, and Gram-positive isolates comprised 60%, and Gram-negative isolates 38%. Among Gram-positive isolates, 10 were *Staphylococcus aureus*, and 11 were Coagulase-negative staphylococci; of *S. aureus *isolates, seven were methicillin-sensitive and three were methicillin-resistant, and of CONS, one isolate was methicillin-sensitive, and 10 isolates were methicillin-resistant. *Enterococci *accounted for three isolates, and *Kokuria kristina* for one isolate. Among Gram-negative isolates, 11 were *Enterobacteriaceae*, and four were non-lactose fermenters. The average susceptibility of Gram-negative isolates was 62% to amikacin and 68% to ciprofloxacin; BL-BLI combinations were 80%, and carbapenems were 87.5% [23]. The single fungal isolate was *Candida parapsilosis* and was fluconazole-susceptible. Mortality was observed in four patients, and crude mortality was 10%. Our study reported a substantially higher CRBSI incidence (10.08 vs. 0.36 episodes per 1,000 catheter-days) and greater in-hospital mortality (31.3% vs. 10%). Although the proportion of Gram-negative isolates was similar (~38-39%) in both studies, Gram-positive organisms predominated in the Mumbai cohort, whereas our study showed a more balanced microbiological profile, likely reflecting differences in patient risk profile, catheter practices, and resource settings.

Shamira Shahar et al. (2021) conducted a retrospective study from January 2016 to December 2018 at UKMMC, in which they enrolled 119 ESRD patients meeting the IDSA criteria for CRBSI or catheter colonization and analyzed 175 events [2]. CRBSI prevalence was 4.2% and catheter colonization 4.8%; the cohort comprised mostly males (59.4%), and 83% used tunneled dialysis catheters. Gram-positive organisms accounted for 62% of isolates, and coagulase-negative staphylococci and methicillin-resistant *S. aureus* emerged as the most frequent pathogens; Gram-negative rods comprised 38%, including *Pseudomonas* and *Enterobacter* species. Clinical presentations and outcomes were similar in CRBSI and colonization groups, and overall mortality was 1.1%, while CRBSI recurrence occurred in 31%. Our study, by contrast, revealed an incidence of 10.08 episodes per 1,000 catheter-days, a higher Gram-negative proportion (23%), a culture-negative rate of 25.4%, and an in-hospital mortality of 31.3%, underscoring regional differences in infection burden and outcomes [2].

Nanyunja et al. (2022) conducted a prospective cohort study from October 2019 to March 2020 at Kiruddu National Referral Hospital and enrolled 121 chronic hemodialysis patients. They observed 41% with at least one CRBSI over 9,441 patient-days for an incidence of 5.2 infections per 1,000 patient-days and reported that 60.3% of isolates were Gram-negative and 36.5% were multidrug-resistant; identified anemia (HR 5.44; p = 0.019) and prior BSI (HR 2.47; p = 0.028) as independent predictors of infection [24]. Our incidence (10.08 per 1,000 catheter-days) exceeds theirs; we found similar Gram-negative predominance but higher culture negativity. Unlike their cohort, catheter dwell-time did not influence risk in our analysis.

In a cohort of 110 patients conducted by Pandit et al. (2024) with tunneled dialysis catheter CRBSI, the overall incidence was 6.9 per 1,000 catheter-days, with a predominance of Gram-negative organisms (64%), including a substantial proportion of carbapenem-resistant isolates (37%), reaffirming the rising importance of Gram-negative pathogens in CRBSI etiology [17]. In contrast to our cohort, where the overall mortality related to CRBSI was 31.3%, Pandit et al. reported a 15% CRBSI-related mortality at three months, with nearly half of survivors experiencing recurrent CRBSI on follow-up. This discrepancy may reflect differences in vascular access type, catheter care protocols, salvage practices, and referral patterns, as well as a broader inclusion of tunneled catheters in their study versus a mix of catheter types in ours.

Findings from our research bear significant implications. The heightened incidence and mortality necessitate an intensified focus on (1) infection-prevention bundles, specifically chlorhexidine dressings, (2) antimicrobial locks, and (3) comprehensive staff training to alleviate early catheter colonization. Empirical antibiotic protocols must encompass prevalent Gram-negative organisms alongside Gram-positives in regions where extended-spectrum beta-lactamase (ESBL) prevalence is documented. While catheter duration did not independently predict mortality, the concentration of infections during the early post-insertion period underscores the importance of proactive fistula planning and timely catheter removal.

We advocate for future investigations to prospectively assess the ramifications of targeted interventions, such as antimicrobial lock solutions or rapid diagnostic assays, on the incidence and outcomes of CRBSIs. Collaborative efforts across multiple centers and regions could facilitate a more comprehensive evaluation of risk determinants and enhance empirical therapy algorithms tailored to local pathogen profiles.

Strengths of the study

Our study employed standardized CDC/IDSA diagnostic criteria within a representative cohort of dialysis patients. We meticulously captured both clinical and microbiological dimensions of CRBSIs and calculated incidence rates utilizing precise catheter-day denominators and focused on clinically meaningful outcomes, such as in-hospital mortality, in a resource-limited dialysis setting.

Limitations of the study

Several limitations were acknowledged in our investigation. The prospective design may have inadvertently introduced selection bias. Furthermore, antibiotic susceptibility patterns were not uniformly accessible for all isolates, thereby precluding a comprehensive resistance profile analysis. The single-centre scope and modest sample size may also affect the generalizability and statistical power of our multivariate models.

## Conclusions

This prospective single-center study demonstrates a substantial burden of CRBSIs among hemodialysis patients in Northeast India, accompanied by considerable in-hospital mortality. The microbiological profile revealed a notable contribution of Gram-negative organisms alongside Gram-positive pathogens, supporting the need for empirical antimicrobial regimens that provide coverage for both groups in similar settings. Although culture positivity and Gram-negative infections were more frequently observed among non-survivors on unadjusted analysis, no clinical or microbiological variable independently predicted mortality after multivariable adjustment, likely reflecting limited sample size and the multifactorial nature of outcomes. These findings emphasize the importance of early AVF creation, strict catheter-care practices, and context-specific infection-prevention strategies. Larger multicenter studies are warranted to inform risk stratification and optimize CRBSI management.
